# Contribution of Vascular Cells to Neointimal Formation

**DOI:** 10.1371/journal.pone.0168914

**Published:** 2017-01-06

**Authors:** Falei Yuan, Dong Wang, Kang Xu, Jixian Wang, Zhijun Zhang, Li Yang, Guo-Yuan Yang, Song Li

**Affiliations:** 1 Department of Neurology, Ruijin Hospital, School of Medicine, Shanghai Jiao Tong University, Shanghai, China; 2 Med-X Research Institute and School of Biomedical Engineering, Shanghai Jiao Tong University, Shanghai, China; 3 Department of Bioengineering, University of California, Berkeley, California, United States of America; 4 Department of Bioengineering, University of California, Los Angeles, California, United States of America; 5 Department of Medicine, University of California, Los Angeles, California, United States of America; 6 Department of Bioengineering, Chong Qing University, Chongqing, China; Duke University, UNITED STATES

## Abstract

The de-differentiation and proliferation of smooth muscle cells (SMCs) are widely accepted as the major contributor to vascular remodeling. However, recent studies indicate that vascular stem cells (VSCs) also play an important role, but their relative contribution remains to be elucidated. In this study, we used genetic lineage tracing approach to further investigate the contribution of SMCs and VSCs to neointimal thickening in response to endothelium denudation injury or artery ligation. *In vitro* and *in vivo* analysis of MYH11-cre/Rosa-loxP-RFP mouse artery showed that SMCs proliferated at a much slower rate than non-SMCs. Upon denudation or ligation injury, two distinct types of neointima were identified: Type-I neointimal cells mainly involved SMCs, while Type II mainly involved non-SMCs. Using Sox10-cre/Rosa-loxP-LacZ mice, we found that Sox10^+^ cells were one of the cell sources in neointima. In addition, lineage tracing using Tie2-cre/Rosa-LoxP-RFP showed that endothelial cells also contributed to the neointimal formation, but rarely transdifferentiated into mesenchymal lineages. These results provide a novel insight into the contribution of vascular cells to neointima formation, and have significant impact on the development of more effective therapies that target specific vascular cell types.

## Introduction

De-differentiation of smooth muscle cells (SMCs) and their major role in intimal thickening after vascular injury have been accepted as a classic theory for decades [1]. In response to vascular injury, media SMCs may de-differentiate, proliferate and migrate into neointima. However, many previous studies use smooth muscle α-actin (ACTA2) as a major marker to identify SMCs [2], which is not accurate because myofibroblasts also express ACTA2. Calponin1 (CNN1) and smooth muscle myosin heavy chain (MYH11) are intermediate and late stage SMC markers respectively, and linage tracing of MYH11^+^ cells offers a better evaluation of SMC’s role in neointima formation [3,4]. However, previous studies using MYH11-cre mice for lineage tracing showed conflicting results [5–7], suggesting that SMCs may not be the only major contributors in the neointima formation.

In the past decade, many studies suggest that vascular stem cells (VSCs) also play an important role in vascular repair and remodeling. For example, we recently identified a Sox10^+^ multipotent VSCs in the media and adventitia of vascular wall and showed that Sox10^+^ cells participated in neointimal formation [5]. Hu *et al*. [8] demonstrated that a group of progenitor/stem cells (Sca-1^+^, c-kit^+^ and Lin^-^) in the adventitia of mouse aorta participated in the development of atherosclerosis following graft transplantation. Sainz *et al*. identified a side population with vascular stem/progenitor properties in the media layer [9]. In addition, a recent study using VE-Cadherin-cre and Tie2-cre mice for lineage tracing revealed that endothelial cells (ECs) contributed to neointimal formation by differentiating into ACTA2^+^MYH11^+^ cells [10].

In present study, we used MYH11-cre/Rosa-loxP-RFP, Sox10-cre/Rosa-loxP-LacZ and Tie2-cre/Rosa-loxP-RFP transgenic mice for lineage tracing, and performed artery denudation injury and ligation experiments to determine the contribution of SMCs, VSCs and ECs to neointimal formation.

## Materials and Methods

### Generation of transgenic mice and genotyping

All the experiments were carried out according to the institutional guidelines and were approved by the Institutional Animal Care and Use Committee of University of California at Berkeley. MYH11-cre (#007742), Tie2-cre (#008863), Rosa-loxP-RFP (#007909) and Rosa-loxP-LacZ (#003474) mice were purchased from The Jackson Laboratory. Sox10-cre mice were a gift from Dr. Andrew S. McCallion and generated as described previously (Stine et al. Genesis, 2009, 47, 765–770). All the male MYH11-cre, Tie2-cre, and Sox10-cre mice were crossed with female Rosa-loxP-RFP or Rosa-loxP-lacZ mice to generate MYH11-cre/Rosa-loxP-RFP, Tie2-cre/Rosa-loxP-RFP, and Sox10-cre/Rosa-loxP-lacZ mice. PCR genotyping was performed according to the protocols provided by The Jackson Lab. The male transgenic mice of two months were used for experiments.

### Cell isolation and culture

Cell isolation methods were described previously [5]. Briefly, adult mouse aortas or arteries were obtained from MYH11-cre/Rosa-loxP-RFP mice and washed 3 times with phosphate buffer saline (PBS) supplemented with 1% penicillin/streptomycin (P/S). Connective tissues and adventitia were carefully removed under a dissecting microscope (Zeiss, Germany). To remove endothelium, vascular tissue was incubated in with 1.5 mg/ml type-II collagenase (Sigma-Aldrich, St. Louis, MO) in Dulbecco’s modified Eagle’s medium (DMEM) for 20 minutes. The tunica media was cut into millimeter-size and placed onto the surface of 6-well plates coated with 1% CellStart (Life Technologies, Grand Island, NY). Cells were cultured in a customized VSC media containing DMEM with 2% chick embryo extract (MP Biomedical, Santa Ana, CA), 1% FBS (Thermo Fisher Scientific, Waltham, MA), 1% N2 (Life Technologies, Grand Island, NY), 2% B27 (Life Technologies, Grand Island, NY), 100 nM retinoic acid (Sigma-Aldrich, St. Louis, MO), 50 nM 2-mercaptoethanol (Sigma-Aldrich, St. Louis, MO), 1% P/S and 20 ng/ml bFGF (R&D Systems, Minneapolis, MN). Cell proliferation was measured using the Click-It 5-ethynyl-2'-deoxyuridine assay (EdU, Life Technologies, Grand Island, NY). For counting the cells, 10 fields of view were taken by microscopy, and EdU^+^RFP^-^ and EdU^+^RFP^+^ cells were counted respectively.

For culture of endothelial cells from Tie2-cre/Rosa-loxP-RFP mice, the carotid artery was harvested and digested in 1.5 mg/ml collagenase for 20 min. The cell suspension was centrifuged at 1000 rpm for 5 min and cell pellet was resuspended in the medium of DMEM supplemented with 10% FBS and 10 ng/ml TGFβ1, and cultured for two weeks.

### Mouse wire injury model and ligation model

The mice of two months old were used for experiments. The mouse carotid artery was subjected to endothelial denudation as described previously [11]. Mice were anesthetized through 1.5% isoflurane inhalation and were supinely placed on a heating pad (Sunbeam 731–500). Target arteries were carefully isolated by blunt separation. For carotid artery wire injury model, a 5–0 nylon suture with a blunt tip was inserted into the external carotid artery and then advanced to the common carotid artery to injure the endothelium. The process of endothelial denudation was repeated three times. Eight MYH11-cre/Rosa-loxP-RFP mice were used for each group in this experiment and common carotid artery was harvested for histological analysis. For femoral artery wire injury model, same suture was inserted into the saphenous artery, and then gently advanced to the iliac artery to induce endothelium denudation [12]. The suture was placed in the artery for 2 minutes and the retreated to induce reperfusion. Three Sox10-cre/Rosa-loxP-LacZ mice were used in this experiment and the femoral artery between saphenous artery and femoral bifurcation was used for histological analysis. Two extra Sox10-cre/Rosa-loxP-RFP mice were used as control.

For carotid artery ligation model, the left common carotid artery was exposed through a small midline incision of the neck. The common carotid artery was completely ligated just proximal to the carotid bifurcation to disrupt blood flow [13,14], and the neointimal formation at the upstream was examined. Six MYH11-cre/Rosa-loxP-RFP mice were used in this experiment and the common carotid artery was used for analysis.

### Immunofluorescence staining and histological analysis

Under deep anesthesia, animals were perfused with normal saline through left cardiac ventricle immediately, followed by 4% paraformaldehyde at pressure of 100 cm H_2_O. Tissue was fixed in 4% paraformaldehyde on ice for 1 hour and then embedded in OCT for cryosectioning. For immunostaining, cells or tissue sections of blood vessels were fixed with 4% paraformaldehyde, permeabilized with 0.5% Triton-100 (Sigma-Aldrich), and blocked with 1% bovine serum albumin (Sigma-Aldrich). Samples were incubated with primary antibodies smooth muscle α-actin (ACTA2, 1:200 dilution, Abcam), calponin-1 (CNN1, 1:200 dilution, Abcam), smooth muscle myosin heavy chain (MYH11, 1:200 dilution, Biomedical Technologies), Sox10 (1:200 dilution, Santa Cruz), Ki67 (1:200 dilution, Abcam) for 2 hours at room temperature, washed with PBS for 3 times, and incubated with appropriate Alexa488-,546-,633- labeled secondary antibodies. Nuclei were counterstained with 4,6-diamidino-2-phenylindole (DAPI). Fluorescence images were collected via a confocal microscopy (Zeiss LSM710). For counting the cells of immunohistological samples, we used the confocal z-stack images with single-cell resolution. For each staining, at least nine sections were used for cell counting.

### Statistical analysis

Data were reported as means±SD. Comparisons among values for all groups were performed by one-way analysis of variance (ANOVA).

## Results

### Proliferation of SMCs and non-SMCs

**Fig 1:**
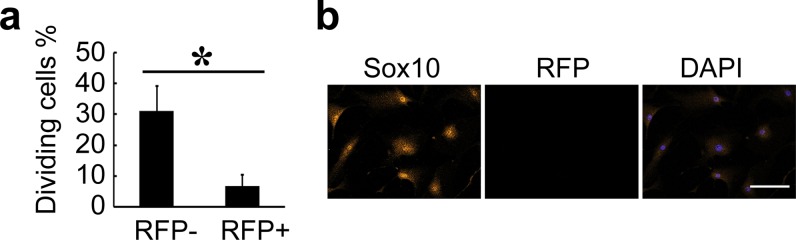
Proliferation of SMCs and non-SMCs. **(a)** The cells were isolated from MYH11-cre/Rosa-loxP-RFP mouse aorta for *in vitro* culture, and the percentage of dividing cells in RFP^-^ and RFP^+^ subpopulations was calculated. Data are presented as mean±SD. One-way ANOVA was used for analysis of significant difference between groups. **p < 0.01. **(b)** The cells were immunostained by the antibody against Sox10. Scale bar, 100 μm.

We previously showed that non-SMCs (e.g., Sox10^+^ cells) in the artery wall could contribute to neointima formation [5]. To compare the proliferation rate of SMCs and non-SMCs, we isolated vascular cells from the aorta of MYH11-cre/Rosa-loxP-RFP mice by enzyme digestion and cultured the cells for two weeks *in vitro*. We found that RFP^-^ cells gradually outgrew RFP^+^ cells in the primary culture. Cell proliferation analysis showed that there were much more dividing RFP^-^ cells than dividing RFP^+^ cells (Fig 1A). Immunostaining showed that a large number of RFP^-^ cells were positive for Sox10 (Fig 1B). This result suggests that non-SMCs such as Sox10^+^ cells may outgrow SMCs and play a more important role than previously thought.

### Heterogeneity of neointimal cells after carotid artery denudation injury

**Fig 2:**
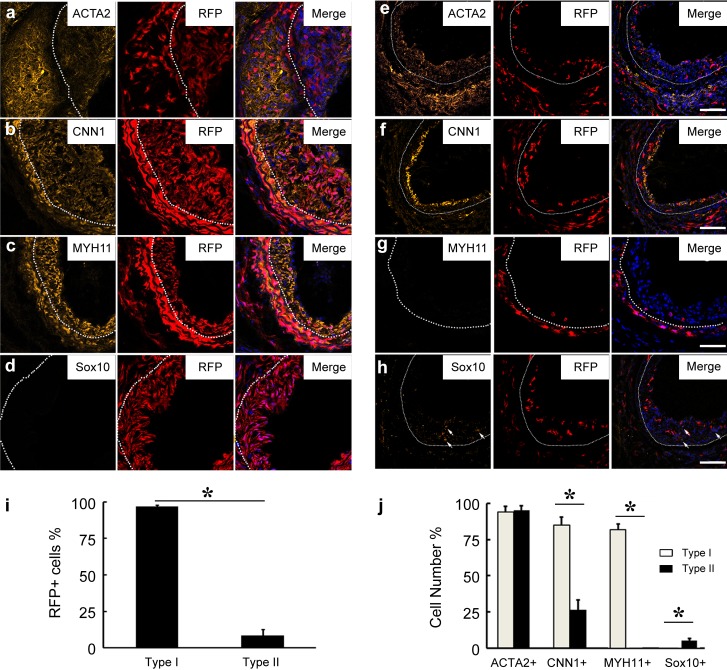
Two types of neointima after carotid artery denudation injury. The cross sections of carotid arteries of MYH11-cre/Rosa-loxP-RFP mice after 2 weeks of denudation injury were immunostained by the antibodies against ACTA2, CNN1, MYH11 and Sox10 (a-h). Dashed lines indicate the border of the neointima. Scale bar, 100 μm. (i) Percentage of RFP+ cells in Type-I and Type-II neointima were calculated. (j) Percentage of ACTA2^+^, CNN1^+^, MYH11^+^ and Sox10^+^ cells in Type-I and Type-II neointima were calculated. Data were presented as mean±SD. One-way ANOVA was used for analysis of significant difference between groups. *p < 0.01.

SMCs have been thought to be the major source of neointimal cells. To investigate relative contribution of SMCs to neointimal formation, we used MYH11-cre/Rosa-loxP-RFP mice to trace SMC fate after two weeks of denudation injury of carotid artery. In the contralateral control carotid arteries, we found all the mice had similar RFP expression in the medial layer with the positive expression of MYH11 (S1 Fig). It was interesting that, in 3 of 8 mice, almost all the neointimal cells of injured arteries were RFP^+^, while in the other 5 mice, the majority of neointimal cells were RFP^-^ (Fig 2A–2I). We defined these two types of neointima as Type I (with abundant RFP^+^ cells) and Type II (with few RFP^+^ cells) respectively.

In Type-I neointima, more than 90% of the RFP^+^ cells were stained positive for SMC markers ACTA2, CNN1 and MYH11 (Fig 2A–2C and 2J), suggesting that these cells may be derived from medial SMCs, or other non-SMCs that differentiated into SMCs upon injury. Few Sox10^+^ cells were found in Type-I neointima (Fig 2D). In contrast, Type-II neointima had more than 90% ACTA2^+^ cells but only about 25% CNN1^+^ cells and rarely MYH11^+^ cells (Fig 2E–2H and 2J). We found ~7% Sox10^+^ cells in the Type-II neointima (Fig 2H). These results suggest that neointimal cells are a heterogeneous population. Sox10^+^ cells may be one of the neointimal cell sources. The low number of Sox10^+^ cells in the immunostaining results may also be explained by the transient expression of Sox10 in activated stem cells.

**Fig 3:**
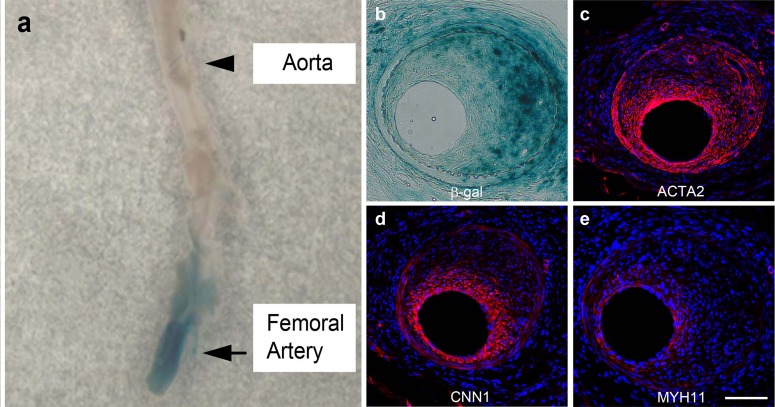
Involvement of Sox10^+^ cells in neointima formation. The femoral arteries of Sox10-cre/Rosa-loxP-LacZ mice were denudated and collected two weeks post-surgery for **(a)** whole-mount β-gal staining, and the cross sections **(b)** were immunostained by the antibodies against ACTA2 (c), CNN1 (d) and MYH11 (e). Scale bar, 100 μm.

To examine Sox10^+^ cell fate during neointima formation, we used Sox10-cre/Rosa-loxP-LacZ or RFP mice for lineage tracing. As SMCs of carotid artery are derived from neural crest [15] and will be labeled by LacZ or RFP during embryonic development of Sox10-cre/Rosa-loxP-LacZ or RFP mice, we performed denudation injury on the femoral artery, which was negative for RFP or LacZ before injury (S2 Fig). After two weeks of injury, we found that a significant percentage of the neointimal cells were β-galactosidase^+^ (β-gal^+^), which expressed ACTA2 and low CNN1 but not MYH11 (Fig 3). This result suggests that Sox10^+^ cells may play an important role during neointimal formation. In addition, β-gal^+^ cells were also found in the medial and adventitial layer and connective tissues around the artery (Fig 3B).

Wire injury may cause inconsistency between individual animals. We noticed that there was a variation of RFP^+^ cell numbers in the medial layers of the injured arteries of different MYH11-cre/Rosa-loxP-RFP mice, which might be caused by over denudation. To reduce this inconsistency, we also performed carotid artery ligation model.

### Two types of neointima were also found after carotid artery ligation

Complete ligation of the blood vessel near the carotid bifurcation blocks the blood flow and induces neointimal formation [14]. This model does not directly injure endothelial and medial layers, which not only avoids the variation in denudation procedure but also allows the study of ECs in neointimal formation.

**Fig 4:**
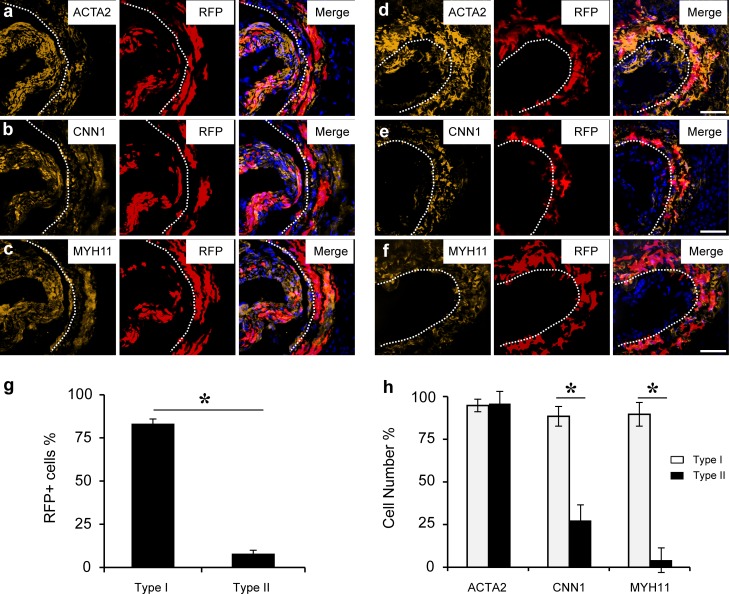
Two types of neointima after carotid artery ligation. The cross sections of carotid arteries of MYH11-cre/Rosa-loxP-RFP mice after 2 weeks of ligation were immunostained by the antibodies against ACTA2, CNN1 and MYH11 **(a-f)** Dashed lines indicate the border of the neointima. Scale bar, 100 μm.

After two weeks of carotid artery ligation in MYH11-cre/Rosa-loxP-RFP mice, we also found two types of neointima similar to the denudation model: 3 of 6 mice had type-I neointima and the other 3 mice had type-II neointima. Most of Type-I neointimal cells were RFP^+^ cells, and expressed ACTA2, CNN1 and MYH11 (Fig 4A–4C), suggesting they were derived from SMCs, or non-SMCs that were activated to differentiate into SMCs; most of Type-II neointimal cells were RFP^-^, expressed ACTA2, but little CNN1 and MYH11 (Fig 4D–4F), suggesting that they are derived from non-SMCs.

### ECs contributed to neointima but did not transdifferentiated into ACTA2^+^ cells

**Fig 5:**
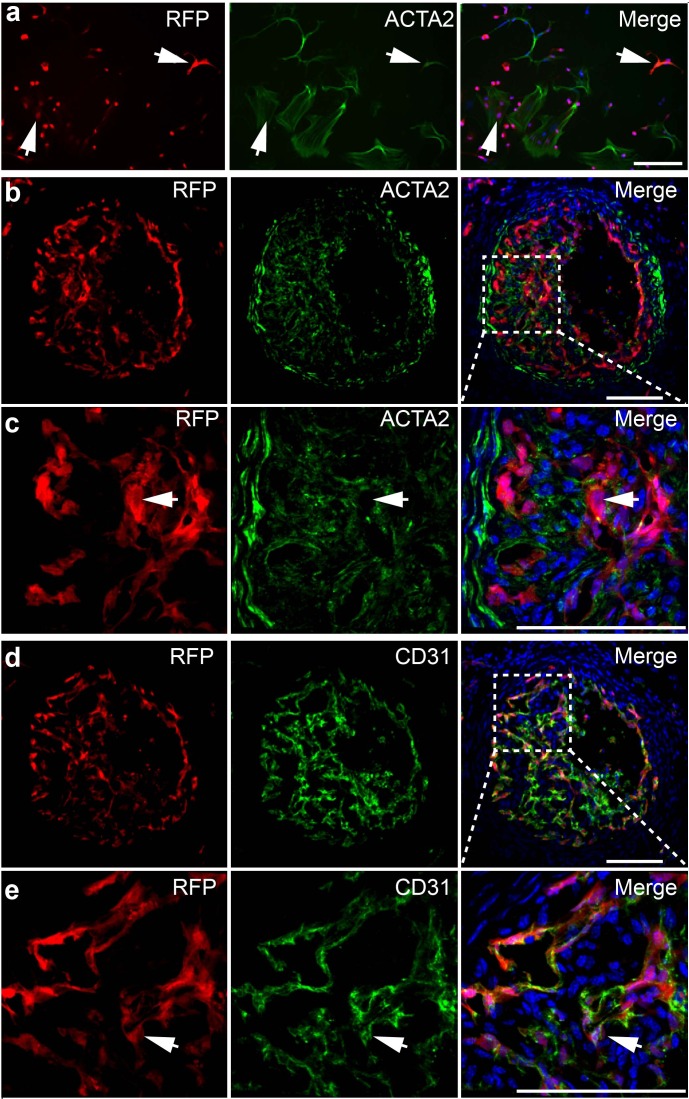
Role of ECs in neointima formation. **(a)** RFP+ cells isolated from Tie2-cre/Rosa-loxP-RFP mice carotid artery were cultured in the medium supplemented with 10 ng/ml TGFβ1 for two weeks, and immunostained by the antibody against ACTA2. **(b-e)** Cross sections of Tie2-cre/Rosa-loxP-RFP mouse carotid artery after two weeks of ligation injury were immunostained by the antibodies against ACTA2 and CD31. Cell nuclei were stained by DAPI. Scale bar, 100 μm.

To examine whether ECs contribute to neointimal formation, we used Tie2-cre/Rosa-loxP-RFP mice to trace endothelial cell fate. We first performed *in vitro* culture experiment. RFP^+^ vascular ECs were isolated and cultured in the medium supplemented with 10 ng/ml TGFβ1. By immunostaining, we found few RFP^+^ ECs expressed ACTA2 under TGFβ1 stimulation (Fig 5A). To investigate whether endothelial-mesenchymal transition existed during neointimal formation *in vivo*, we performed carotid artery ligation model on Tie2-cre/Rosa-loxP-RFP mice. We found that ECs showed an invasive phenotype in neointima (Fig 5B–5E). Significant number of ECs were found in the neointima, but most of them did not express ACTA2 (Fig 5B and 5C). These RFP^+^ cells still expressed the EC marker CD31 (Fig 5D and 5E), suggesting that ECs still maintained EC phenotype and did not trans-differentiate into ACTA2^+^ cells *in vivo*.

## Discussion

There has been controversy about the origins of neointimal cells. Historically, fibroblasts or fibroblast-like cells were thought to be the major cell types of arterial neointima [16]. However a different opinion is that SMCs are the only cells that exist in arterial media and contribute to neointimal formation [17]. Using a transgenic mouse model that expressed inducible creERT2 under the control of MYH11 promoter, Herring *et al*. found that medial SMCs are the major cells contributing to neointimal formation [6]. However, arterial media layer was reported to be composed of heterogeneous populations of cells including SMCs that expressed MYH11 and ACTA2, and non-SMCs that didn’t express these markers [9,18]. Heterogeneity also exists in the SMC population and only a subpopulation among them was reported to contribute to neointima formation [7]. In recent years, adventitial stem cells have been reported to contribute to neointimal formation [8]. Our previous study found a stem cell population in arterial medial layer that expressed markers of neural crest stem cells and mesenchymal stem cells, and could be a source of neointima [5,19]. In this study, we used two different vascular injury models and investigated the contribution of SMCs, Sox10^+^ VSCs and ECs to neointimal thickening. We show that Sox10^+^ VSCs and SMCs are two distinct populations, and for the first time, defined two types of neointima. Type-I neointimal cells expressed mature SMC markers and may be derived from medial SMCs or other non-SMCs. However, Type-II neointima involves mostly other cell types, which is consistent with the recent report [5] and suggests that SMCs are not the major cell type in some neointima formation in vascular injury models. The underlying mechanisms that result in these two types of neointima are unknown. We postulate that different extents of vascular injury in endothelium, elastic lamina and SMCs and the inflammatory responses may activate different cell types and that the presence and proximity of cell types near the lesion sites may modulate the relative contribution of vascular cells to the neointimal formation. This finding, if verified in human diseased vessels, will have profound impact on the development of new therapies that target specific cell types. Given the complex genetic background and the various causes of vascular diseases in human, it is likely that multiple cellular sources contribute to neointimal formation.

Our data suggest that Sox10^+^ VSCs can be an important source of SMA^+^ cells in neointima. Sox10 is specifically expressed in neural crest stem cells during embryonic development, which maintains the multipotency of neural crest stem cells [20]. It is also reported as an important marker for maintaining neural crest-like cells in an undifferentiated state [21]. However, Sox10^+^ VSCs in adult tissues may or may not be related to the neural crest cells during the development. In normal artery, the number of Sox10^+^ VSCs is low and they are located in the media and adventitia layer of arteries [5,19]. Upon vascular injury, Sox10^+^ VSCs can proliferate and migrate [19], thus participating in vascular remodeling. The mechanisms of VSC activation and multiplication in response to injury and inflammatory signals remain to be investigated. It is possible that the involvement of VSCs in neointimal formation may be regulated by the microenvironmental factors and the location and abundance of VSCs near the lesion sites.

Ligation model does not directly injure endothelium and SMCs in the vessel, which may be difficult for SMCs to migrate into the neointima when elastic lamina is not damage. However, SMCs were still found in the neointima possibly due to the remodeling process upon ligation [22,23]. We show that ECs also play an important role in the neointima formation in the ligation model where endothelium is not denuded. Although there is evidence that ECs may trans-differentiate into mesenchymal cells in neointima, we find this a low frequency event *in vivo* and *in vitro*. It is likely that EndMT requires additional signals and may only happen under specific conditions.

In summary, we have demonstrated that SMCs, Sox10^+^ VSCs and ECs all significantly contribute to the neointima formation, and their relative contribution may depend on many factors in the vascular niche. It is evident that SMCs are not the only major cell type in neointima formation. These findings will help develop new strategies to finally cure vascular diseases.

## Supporting Information

S1 Fig Normal (control, without injury) carotid arteries of MYH11-cre/Rosa-loxP-RFP mice were cryosectioned and immunostained by the antibody against MYH11. Similar RFP expression was observed in all of the mice used in the experiment. Cell nuclei were stained by DAPI. Scale bar, 100 μm.
(PDF)

S2 Fig Normal (control, without injury) femoral arteries of Sox10-cre/Rosa-loxP-RFP mice were cryosectioned and immunostained by the antibody against CD31. RFP only labeled femoral nerve (arrow), but not the cells in the wall of femoral artery. Cell nuclei were stained by DAPI. Scale bar, 100 μm.
(PDF)

## Abbreviations

CNN1: Calponin1
EC: endothelial cell
EdU: 5-ethynyl-2'-deoxyuridine
MYH11: smooth muscle myosin heavy chain
ACTA2: smooth muscle α-actin
SMC: smooth muscle cell
VSC: vascular stem cell
RFP: red fluorescent protein
