# Supplementary material for: Contribution of Vascular Cells to Neointimal Formation
Source: PLoS One. 2017 Jan 6;12(1):e0168914. doi: 10.1371/journal.pone.0168914 (PMC5218548; doi:10.1371/journal.pone.0168914)
Supplement: S1 Fig — Similar RFP expression was observed in all of the mice used in the experiment. Cell nuclei were stained by DAPI. Scale bar, 100 μm. (PDF) [file pone.0168914.s001.pdf]

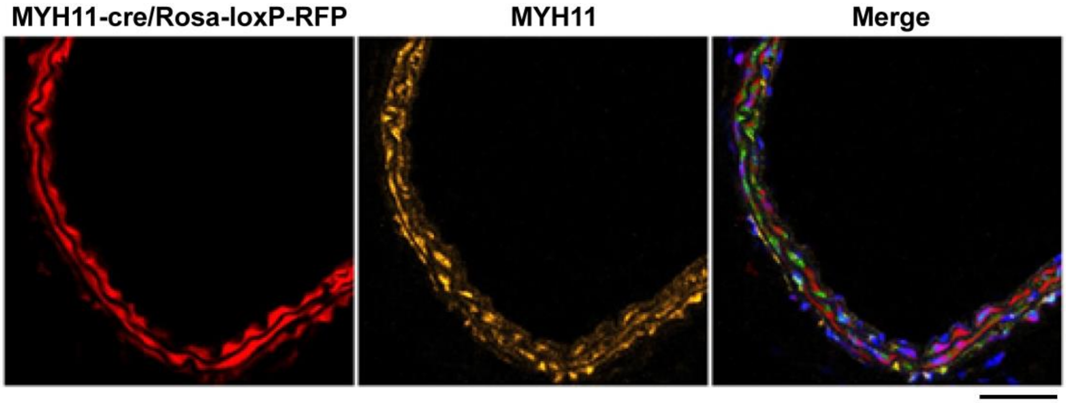

S1 Fig. Normal (control, without injury) carotid arteries of MYH11-cre/Rosa-loxP-RFP mice were cryosectioned and immunostained by the antibody against MYH11. Similar RFP expression was observed in all of the mice used in the experiment. Cell nuclei were stained by DAPI. Scale bar, 100 µm.
