# Supplementary material for: Contribution of Vascular Cells to Neointimal Formation
Source: PLoS One. 2017 Jan 6;12(1):e0168914. doi: 10.1371/journal.pone.0168914 (PMC5218548; doi:10.1371/journal.pone.0168914)
Supplement: S2 Fig — RFP only labeled femoral nerve (arrow), but not the cells in the wall of femoral artery. Cell nuclei were stained by DAPI. Scale bar, 100 μm. (PDF) [file pone.0168914.s002.pdf]

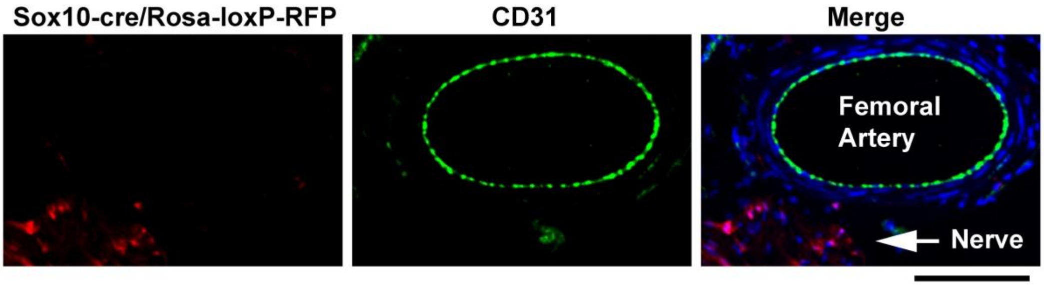

S2 Fig. Normal (control, without injury) femoral arteries of Sox10-cre/Rosa-loxP-RFP mice were cryosectioned and immunostained by the antibody against CD31. RFP only labeled femoral nerve (arrow), but not the cells in the wall of femoral artery. Cell nuclei were stained by DAPI. Scale bar, 100 μm.
